# Dissecting the Dual Nature of Hyaluronan in the Tumor Microenvironment

**DOI:** 10.3389/fimmu.2019.00947

**Published:** 2019-05-10

**Authors:** Muhan Liu, Cornelia Tolg, Eva Turley

**Affiliations:** ^1^Department of Biochemistry, Western University, London, ON, Canada; ^2^London Regional Cancer Program, Lawson Health Research Institute, London, ON, Canada; ^3^Department of Oncology, Biochemistry and Surgery, Schulich School of Medicine and Dentistry, Western University, London, ON, Canada

**Keywords:** hyaluronan, hyaluronan receptors, tumor microenvironment, cancer resistance, tumor initiation, CD44, RHAMM

## Abstract

Hyaluronan (HA) is a glycosaminoglycan with a simple structure but diverse and often opposing functions. The biological activities of this polysaccharide depend on its molecular weight and the identity of interacting receptors. HA is initially synthesized as high molecular-weight (HMW) polymers, which maintain homeostasis and restrain cell proliferation and migration in normal tissues. These HMW-HA functions are mediated by constitutively expressed receptors including CD44, LYVE-1, and STABILIN2. During normal processes such as tissue remodeling and wound healing, HMW-HA is fragmented into low molecular weight polymers (LMW-HA) by hyaluronidases and free radicals, which promote inflammation, immune cell recruitment and the epithelial cell migration. These functions are mediated by RHAMM and TLR2,4, which coordinate signaling with CD44 and other HA receptors. Tumor cells hijack the normally tightly regulated HA production/fragmentation associated with wound repair/remodeling, and these HA functions participate in driving and maintaining malignant progression. However, elevated HMW-HA production in the absence of fragmentation is linked to cancer resistance. The controlled production of HA polymer sizes and their functions are predicted to be key to dissecting the role of microenvironment in permitting or restraining the oncogenic potential of tissues. This review focuses on the dual nature of HA in cancer initiation vs. resistance, and the therapeutic potential of HA for chemo-prevention and as a target for cancer management.

## Introduction

Cancer is the second leading cause of mortality worldwide according to GLOBOCAN estimates, accounting for ~9.6 million death in 2018 (1). Two decades ago, Hanahan et al. proposed the six classic hallmarks during the multi-step development of cancer (sustained proliferative signaling, evasion of growth suppressors, limitless replicative potential, resistance of apoptosis, sustained angiogenesis, and invasion/metastasis) (2). Conceptual advancements in the past two decades have added evasion of immuno-surveillance, elimination of cell energy limitation, genome instability, and the participation of host cells in facilitating tumor initiation and progression (3, 4). Emphasis has historically been placed on the key role of mutations and genomic instability as drivers of tumor initiation and progression. However, the somatic mutation burden in physiologically normal tissues can approach the level of many cancers (5). These somatic mutations increase with age, and include cancer driver genes, which appear to be under strong positive selective pressure. They occur in the absence of evidence for tumorigenic conversion (5), suggesting that driver gene mutations by themselves are insufficient for cancer initiation. Experimental data also predict that the microenvironment can block or permit the oncogenic potential of mutations (6–9). For example, teratocarcinomas transplanted into early embryos do not form tumors but participate in the development of normal tissues (10). Moreover, the tumorigenic potential of genomically unstable breast cancer cell lines can be curtailed by blocking specific signaling pathways from the extracellular matrix (11–13) suggesting the paracrine interactions between the host cells, tumor cells, and the extracellular matrix can disable or enable the oncogenic potential of mutations. Therefore, dissecting the nature of such interactions is critical to identify therapeutic targets that manage tumor initiation, tumor progression, and post-treatment tumor recurrence.

**Figure 1:**
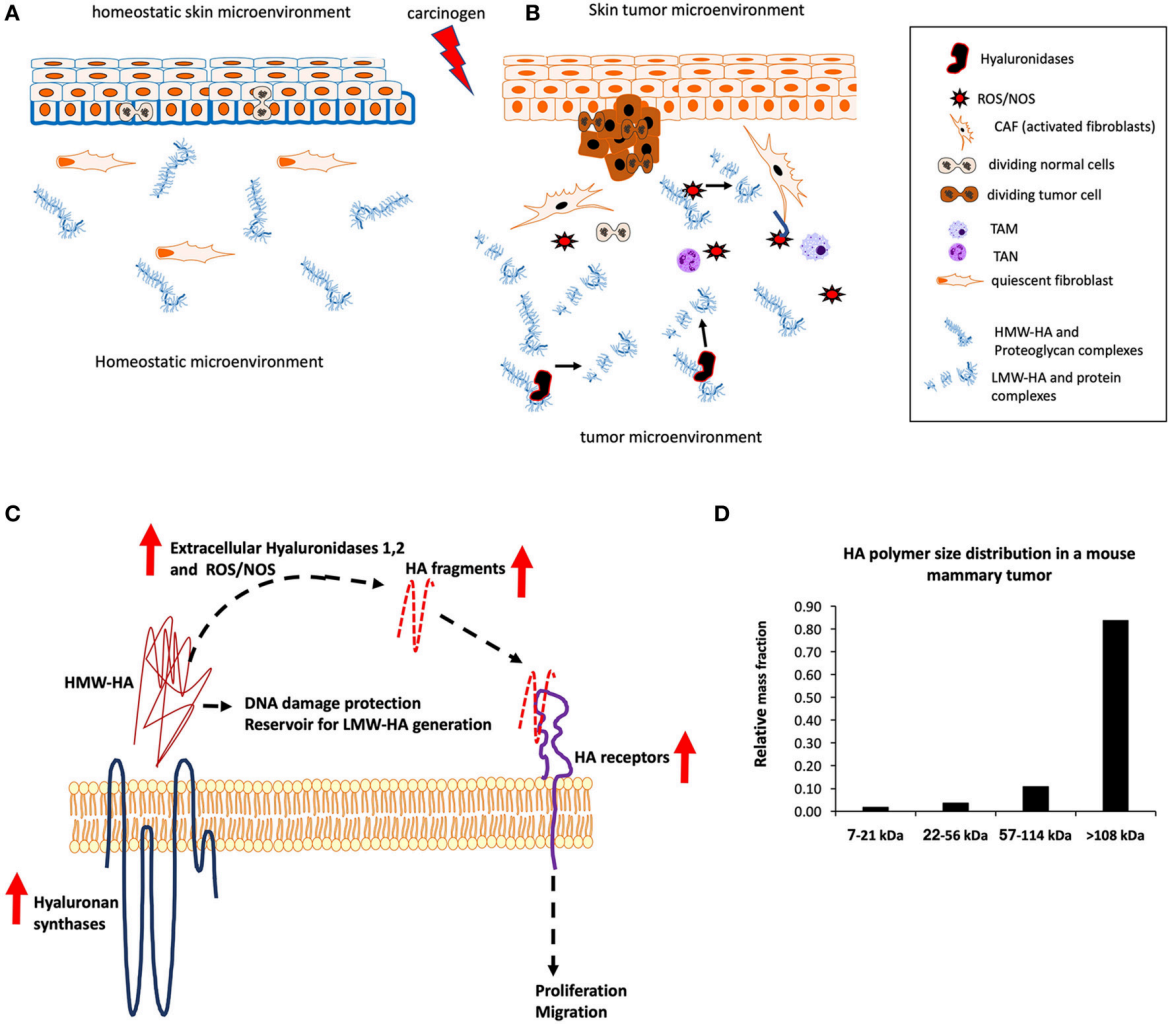
Hyaluronan synthesis and fragmentation in normal and tumor microenvironments. **(A)** Homeostatic skin is characterized by an organized epidermis with the regulated symmetric and asymmetric division of basal keratinocytes. These are covered by layers of HMW-HA coats (blue outline). Dermal fibroblasts are quiescent and HMW-HA is organized into complexes with proteins and proteoglycans. Extracellular LMW-HA accumulation is restricted. **(B)** Tumor-initiating events result in disorganized growth of epidermal cells and changes to HA organization and processing. HA synthesis increases, hyaluronidases are expressed/released and reactive oxygen/nitrogen species (ROS/NOS) production is high, resulting in HA fragmentation and reduced organization of macromolecular complexes. HMW-HA coats around epidermal cells are reduced. LMW-HA activates fibroblasts (CAFs, cancer-associated or activated fibroblasts) and attracts immune cells [tumor-associated neutrophils (TANs) and tumor-associated macrophages (TAMs)] that produce ROS/NOS. The fragmented and disorganized tumor microenvironment supports tumor proliferation and evasion of immune surveillance. **(C)** In disease states such as tumors or chronically inflamed tissues, native or HMW-HA synthesis is increased by constitutively elevated HAS expression. LMW-HA accumulates due to increased expression and activity of extracellular hyaluronidase activity and reactive oxygen/nitrogen species (ROS/NOS) produced by stressed tissues. LMW-HA activates pro-migratory and proliferation pathways through CD44, RHAMM and TLR2,4, whose expression is also increased. In a disease such as cancer, HMW-HA contributes to a stem cell-like microenvironment that is immuno-suppressive and may protect tumor cells from DNA damage. **(D)** The continued accumulation of HMW HA polymers also provides a source for generating LMW-HA that accumulates in tumor microenvironments as shown in **(A)**. These fragments can be targeted by peptide mimetics that bind and sequestering them, preventing their activation of pro-migration and proliferation signaling pathways.

The tissue polysaccharide, hyaluronan (HA), is one example of an extracellular matrix component that participates in cancer initiation and progression (5, 14–16). HA, like many factors, is multifunctional and has both tumor-promoting and -suppressing properties. HA is an anionic, linear, non-sulfated glycosaminoglycan (GAGs) composed of repeating disaccharide units of glucuronic acid, and N-acetylglucosamine that are synthesized by hyaluronan synthases (HAS1-3) and that can achieve molecular weights in excess of 1,000 kDa. Fragmentation of these large polymers by free radicals and hyaluronidases (HYAL1-3) generate low molecular-weight HA varying from 1 to 500 kDa. Despite its simple primary chemical structure, the large range of polymer sizes generates diverse physiochemical functions and signaling properties (14, 15). High molecular weight HA (HMW-HA, defined here as >*500 kDa* and in text) predominates in normal tissues, where it provides a scaffold for protein interactions and is essential in maintaining tissue homeostasis. The viscoelastic properties of HMW-HA contribute to porosity and malleability of extracellular matrices (e.g., stem cell niches) (17–20), which are important for resistance to somatic mutation, protection against mechanical damage, and regulating cell trafficking. HMW-HA is also anti-inflammatory and anti-proliferative, which may contribute to tumor resistance in normal tissues (21–24). Fragmentation of HMW-HA into low molecular-weight polymers (LMW-HA, defined here as 7–200 kDa, Figures 1A–D and in text) is minimal or absent in homeostatic tissues, but is increased during response-to-injury and remodeling events in the embryo and adult (25, 26) due to expression of HYAL1-3 and generation of ROS/NOS. LMW-HA activates signaling cascades that promote cell migration, proliferation, immune cell influx, and mesenchymal cell trafficking (27, 28) (Figures 1A–C). This review highlights the consequence of these opposing functions of HA polymer sizes to tumor resistance, initiation and progression. The potential of HA polymers and the processing/signaling machinery for these polymers as therapeutic targets in cancer prevention and management is emphasized.

## The Hyaluronome

HA is uniquely synthesized by plasma membrane HAS. Polymers are initiated on the cytoplasmic face of these proteins, then extruded through pores created by aggregated HAS into the ECM (29–32). The three HAS isoforms are encoded on different chromosomes and exhibit distinct tissue distribution and enzymatic properties (31, 33, 34). HA polymers are degraded by three major hyaluronidases (HYAL1-3). HYAL1 and 3 are primarily located in the lysosome and, together with glucosaminidases and glucuronidases (35), degrade HA polymers into monomers. HYAL2 is located at the cell surface, which together with extracellular reactive oxygen/nitrative species (ROS/NOS), generate extracellular LMW-HA (36). These HMW- and LMW-HA polymers bind to CD44, RHAMM, LYVE1, TLR2/4, STAB2, and LAYILLIN (37). HA receptors lack kinase activity and signal by associating with growth factor receptors (e.g., EGFR, PDGFR, and TGFBR) (38, 39). One function of HA:HA receptor interactions is to regulate receptor clustering and membrane localization (40). These critical HA functions are mediated by polymer size and the size preference of HA receptors. For example, while CD44, RHAMM and TLR2,4 bind to a range of sizes, RHAMM and TLR2,4 preferentially bind to very low molecular-weight HA (<7 kDa) (4, 37). The functional complexity of HA polymer signaling is additionally generated by the extensive and often tissue/stimulus-specific alternative splicing of CD44 (41, 42), tissue-specific expression of LYVE1 (lymphatics), and STAB2 (liver) (43–46). The multi-compartmentalization, stress- and stem cell-specific expression of RHAMM, as well as cell context-dependent interaction of HA receptors with each other (e.g., RHAMM/CD44, RHAMM/TLR2,4) also influence HA-mediated signaling (37, 38).

How this HA signaling machinery functions in tumorigenesis can be complex and unpredictable. For example, CD44 can perform tumor-promoting or -suppressing functions depending on its association with protein partners (47). Despite its elevated expression on progenitor-like tumor-initiating cells, deletion of CD44 in a mouse model of luminal breast cancer susceptibility increases rather than decreases metastases. Similarly, either increasing or decreasing intracellular RHAMM expression will de-regulate its functions in centrosome and spindles, increasing the potential for generating genomic instability and enhancing tumorigenesis (48). Such dual properties make direct therapeutic targeting of HA receptors challenging. Nevertheless, de-regulated HA metabolism/signaling typifies many cancers and often predicts poor clinical outcome (49–56). Therefore, developing an understanding of HA metabolism/signaling as a microenvironmental factor that contributes to cancer susceptibility, initiation and progression is likely to identify therapeutic avenues for managing this disease.

## Hyaluronan: a Double-Edged Sword in Cancer?

The tumor microenvironment is emerging as a critical factor in cancer progression and recurrence. HA production and accumulation in both the stroma and tumor parenchyma is characteristic of prostate, bladder, lung, breast and other cancers, and is linked to poor clinical outcome (49, 51, 52, 57–59). The majority of these studies do not report the molecular-weight ranges of HA present in the tumor samples. However, the increased expression of HA receptors that preferentially bind to LMW-HA (e.g., RHAMM), increased HYAL expression (e.g., HYAL1,2) (49, 50, 55, 57, 60, 61), and elevated ROS levels predict that LMW-HA is elevated. However, It is likely that both HMW and LMW-HA are increased in tumor microenvironments and that both contribute to tumor survival, growth and aggression (Figures 1B,C). Data mining using TCGA data sets in the cBioPortal for Cancer Genomics (www.cbioportal.org) provides unbiased evidence the association of increased HA production and fragmentation with cancer patient survival. For example, increases in hyaluronidases, HAS and HA receptors are significantly linked to reduced survival in colorectal (e.g., HAS2, HMMR, CD44, HYAL1, *p* = 0.013) (62), and breast [e.g., HAS2, HMMR, HYAL2, *p* = 0.023, (63) and *p* = 5.24e-3(64)] cancers.

Experimental studies provide direct evidence for the importance of elevated HA production in tumor initiation and progression. As examples, MMTV-Neu mice over-expressing a HAS2 transgene exhibit increased mammary tumor incidence and growth compared to wildtype animals (65). HAS2-mediated acceleration of tumor progression results in part from resistance to apoptosis caused by constitutive activation of PI3K/AKT signaling (65, 66). Conversely, HAS2 knockdown in metastatic breast cancer cell lines arrests tumor cells in G0/G1 as a result of reduced cyclin A, B, and cdc2 expression (67). Stable knockdown of this HAS in MDA-MB-231 breast cancer cells decreases their invasion, which is rescued by HAS2 re-expression (66). Conversely, cell sorting of breast tumor cell lines that bind to high levels of HA identifies highly invasive, metastatic subpopulations (68). Experimental evidence from these types of studies suggests that elevated HMW-HA accumulation in either the peri-tumor stroma or tumor parenchyma performs several key functions that favor tumor growth in addition to protection from apoptosis. These include reducing uptake of chemotherapeutic drugs by regulating drug transport proteins, reducing neo-angiogenesis, and reducing drug diffusion into the tumor by increasing local tissue hydrostatic pressure (69–71). HMW-HA reduces immune surveillance at least in part by blocking neo-angiogenesis. Importantly, abundant HMW-HA polymers also provide a stable source for generating LMW-HA.

An example of evidence supporting this latter function is provided by the enhanced invasion of tumor cells when they are transfected with both HAS2 and hyaluronidases (72). HYAL1 knockdown reduces tumorigenicity of breast cancer cell lines (73), while forced expression of HYAL1 promotes tumor cell growth and migration in culture and *in vivo* (74, 75). LMW-HA polymers promote neo-angiogenesis, tumor cell migration, invasion, and proliferation (16). LMW-HA also attracts macrophages, which polarize into subpopulations that protect tumor cells from adaptive immune cell killing (76). This fragment-specific signaling is mediated by HA receptors, the best studied of which are RHAMM and CD44. Tumor cell migration stimulated by LMW-HA is often associated with an epithelial-mesenchymal transition mediated by CD44 through activating PI3K/AKT and TGFβ signaling (65), while RHAMM is linked to increasing tumor cell migration via regulating ERK (77–79) and AURKA/beta-catenin pathways (80, 81). This signaling not only impacts the functional properties of tumor cells but also regulates stromal cells properties. Thus, LMW-HA/CD44/RHAMM binding promotes angiogenic capillary invasion into the tumor microenvironment. In MMTV-Neu mice, elevated LMW-HA production induces intra-tumoral microvessel formation through promoting angiogenic factor secretion, and enhanced type I collagen, fibronectin, bFGF, CD31 mRNA expression (65, 74). Further, bFGF-induced angiogenesis is accelerated in the presence of LMW-HA (74).

LMW-HA in the tumor microenvironment also promotes monocyte recruitment and differentiation of cytotoxic M1 subtypes into the M2 subtype by altering the Th1/Th2 cytokine balance, thereby enhancing local ROS level, which increases LMW-HA generation and M2 monocyte accumulation (76). This appears to have clinical relevance since both increased M2-like tumor-associated macrophages (TAMs) and LMW-HA accumulation correlate with tumor metastatic potential and poor prognosis in breast cancer patients (54). Both CD44 antibodies and LMW-HA binding peptides reduce monocyte activation and M2 polarization predict that these effects are modulated by LMW-HA:CD44 interactions(76). Moreover, tumor-derived LMW-HA promotes tolerance of tumor cells to infiltrating macrophages (82). This tumoricidal neutralization is suggested to involve IRAK-M (interleukin-1 receptor-associated kinase M), with LMW-HA as an extracellular modulator through both TLR2,4 and CD44 (82).

Collectively, clinical and experimental studies show the importance of tumor or peri-tumor stromal cell HA production and fragmentation for tumor progression. Targeting HA may therefore provide promising therapeutic approaches in cancer management and treatment.

## Therapeutic Approaches for Reducing HA Production and Fragmentation to Control Tumorigenesis

### Inhibiting HA Synthesis

4-Methylumbelliferone (4-MU) is an inhibitor that depletes one of the building blocks (glucuronic acid, GA) of HA synthesis (83, 84). In 4-MU treated mammalian cells, UDP-transferase catalyzes the transfer of GA onto 4-MU, thus depleting the pool of cytoplasmic UDP-GA and inhibiting HA synthesis (83, 85). 4-MU also decreases HAS2/3 expression (60–80% in cancer cell lines) (86). This suppression is accompanied by reduced CD44 and RHAMM expression, suggesting a feedback loop between HA synthesis and receptor expression (86). HA-mediated downstream signaling is therefore inhibited following 4-MU administration with a consequent reduction in proliferation, migration, and invasion of cancer cells. 4-MU reduces metastasis in skin, lung, osteosarcoma, and breast cancer xenograft models(85, 87–91). Dietary incorporation of 4-MU is also associated with enhanced chemo-prevention: daily ingestion of 4-MU (450 mg/kg) for 28 weeks abrogated prostate tumor initiation and metastasis in experimental models (91). Nevertheless, this approach is not specific for tumor-associated HA and would also block the desirable functions of HMW-HA in normal tissues.

### Targeting LMW-HA

#### Blocking Hyaluronidase Activity

Natural derivatives (i.e., heparin, glycyrrhizic acid, and sodium aurothiomalate) and drugs (i.e., fenoprofen) targeting HYAL isoforms have been investigated as therapeutic agents (26, 85, 88). Originally described as urinary HYAL inhibitors, O-sulfated HA derivatives (sHA) exhibit potent inhibition of HYAL1 activity (IC_50_: 0.0083–0.019 μM) through binding to allosteric sites (92, 93). Moreover, sHA exhibits more effective non-competitive than competitive inhibition, suggesting its efficacy would not be impeded by elevated HA concentration in tumor tissues. In a prostate cancer animal model, sHA-application inhibited HYAL1 activity and triggered apoptosis through the extrinsic pathway (70). The downregulation of HYAL1 correlated with reduced CD44 and RHAMM expression, and PI3K/AKT signaling, suggesting a feedback mechanism between HA degradation and signaling in tumor cells. The proliferative and invasive capacities of prostate cancer cells are also decreased significantly after sHA-treatment (69).

Conversely, increased degradation of stromal HA by PEGPH20 (a pegylated hyaluronidase) in pancreatic cancer degrades the HA capsule that accumulates around these tumor cells to allow better exposure of tumor cells to chemotherapy and release suppression of neo-angiogenesis (94). PEGPH20 efficacy in facilitating a response of pancreatic tumor cells to chemotherapy is currently being assessed in Phase 3 clinical trials for metastatic pancreatic ductal adenocarcinoma (ClinicalTrials.gov) (94). PEGPH20 administration in animal models of pancreatic cancer induced vascular collapse in tumors and dramatically elevated the interstitial fluid pressure, enhancing perfusion, and delivery of chemotherapeutic drugs into the solid tumor (71). Thus, combined administration of PEGPH20 and other chemotherapy (i,e: gemcitabine) promoted survival and reduced tumor growth in mouse models through inducing apoptosis and suppressing proliferation (71).

#### Sequestering HA Fragments

The use of peptides or small molecules that bind to the HA binding regions of HA receptors, such as CD44 and RHAMM, is another therapeutic approach that attempts to sequester LMW-HA, thus preventing these from interacting with receptors to abrogate signal activation. To date, extensive use of peptides to block tumorigenesis has not yet been reported. However, RHAMM- or HA-binding peptides can inhibit invasion and survival of prostate and breast cancer cells in culture and *in vivo* (95, 96). LMW-HA binding peptides have been isolated by screening phage display libraries (97, 98) and by designing RHAMM-like peptides that bind to LMW-HAs with nM affinity (95). These have been shown to block inflammation and fibrosis in both cell culture and animal models (95). Since some cancers originate from progenitor-like cancer-initiating cells, which commonly express CD44, small molecule inhibitors of CD44:HA binding are also being developed (99). However, polysaccharide:protein interactions in general and HA-CD44 binding in particular occur over large surfaces, making design of small molecule inhibitors challenging (95). The amounts of LMW-HAs that accumulate in the peri-tumor stroma is surprisingly small (Figure 1D). These low amounts predict the use of LMW-HA binding peptides may be one of the most effective approaches for targeting the tumorigenic properties tumor microenvironments.

## HMW-HA, Hyaluronidase, and CD44 as Tumor Prevention Mechanisms

HMW-HA can also suppress tumor initiation in cancer-prone species by inhibiting proliferation, migration/invasion and ROS-mediated DNA damage. HMW-HA blocks proliferation by signaling through CD44 to promote G1/G0 arrest (100) and, as noted above, alter tumor growth kinetics by suppressing neo-angiogenesis and immune responses (101). Consistent with these reported functions, HMW-HA suppresses growth of murine astrocytoma cell lines, glioma and colon carcinoma xenografts. HMW-HA has also been reported to reduce the migratory and invasive capacity of aggressive cancer cells (23, 102), which could occur by several mechanisms. These include: (1) HMW-HA strengthens cell-cell junctions and decreases the permeability of ECM, thus preventing invasion (103); (2) HMW-HA promotes tight junction formation and myosin polymerization in lymphatics by displacing LMW-HA from the LYVE-1 receptor, which restricts invasion into lymph nodes (103); (3) HMW-HA blocks migration by enhancing NF2 and CD44 co-association, which activates the tumor suppressor properties of CD44 (24); and (4) HMW-HA modifies the motogenic effects of growth factor signaling. As an example, bFGF-treatment decreases sarcoma cell migration as a result of increased HAS1, HAS2, and CD44 expression combined with a reduced expression of HYAL2 (74), which favors accumulation of HMW-HA(55). Further, the addition of HMW-HA reduced fibrosarcoma cell migration, which can be overridden by exogenous LMW-HA or HAS1 knockdown (74). In addition to these growth- and migratory-suppressing effects, HMW-HA also reduces ROS-mediated DNA damage, which lessens mutational burden and thereby reduces the risk of neoplastic transformation (36, 104).

While the above studies predict functions of HMW-HA that could contribute to tumor resistance in cancer-prone species, such as mice and humans, the cancer-resistant naked mole-rat (*Heterocephalus glaber*) provided the first direct evidence for HMW-HA in suppressing carcinogen-induced tumorigenesis(24, 105). In *H. glaber*, the combination of HAS2 overexpression, strongly reduced hyaluronidase activity, and signaling through CD44 provides resistance to several carcinogenic insults, including UVB and activated RAS (24). Interestingly, HMW-HA accumulates in some tissues, notably skin, and remains high throughout the lifetime of this animal (24). In contrast, HMW-HA declines in skin and most tissues of human and mouse with age (106–110) and after chronic exposure to UVB, which is the predominant carcinogen causing keratinocyte tumors.

**Figure 2:**
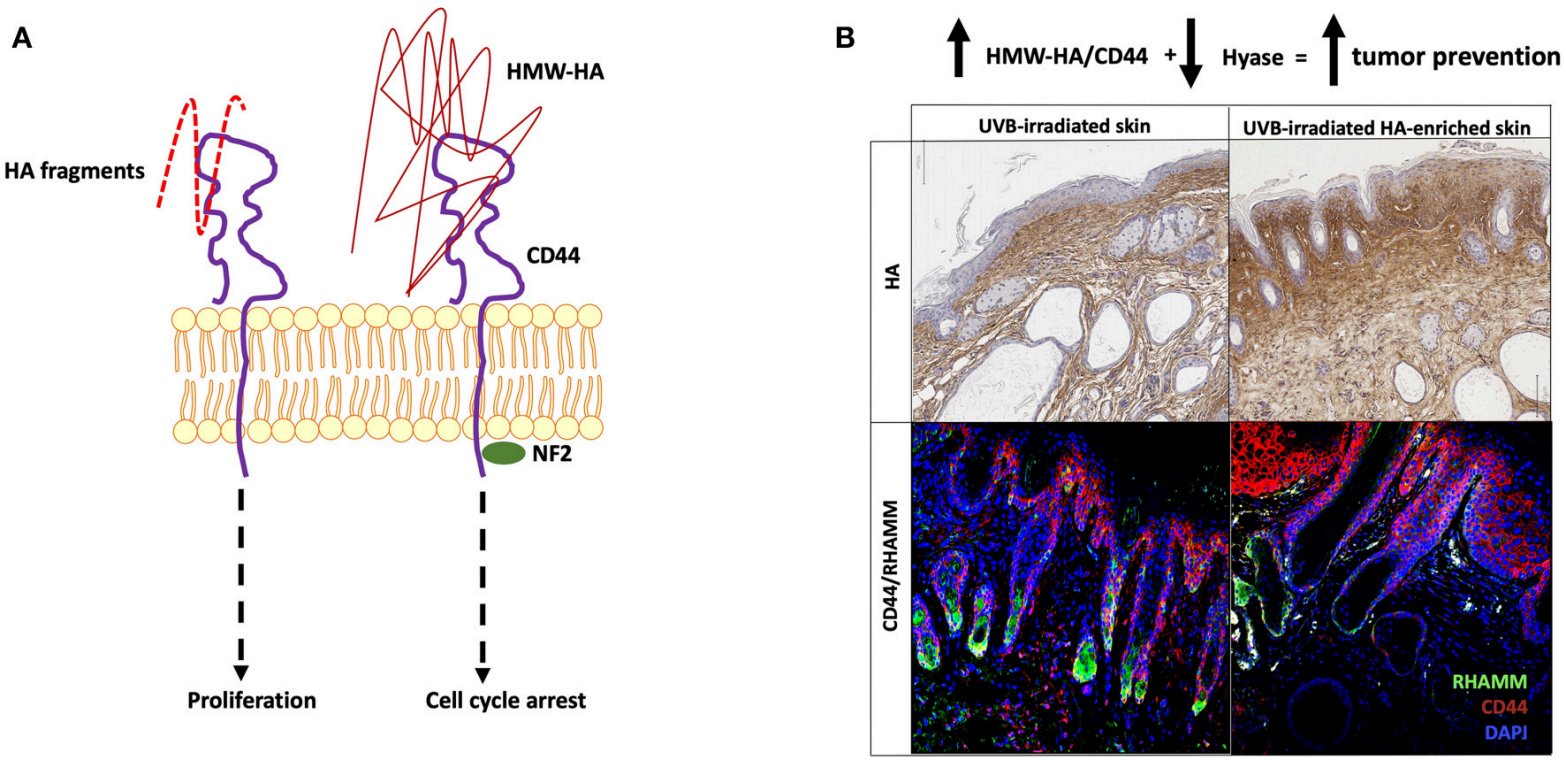
HMW-HA functions in tumor prevention. **(A)** LMW-HA:CD44 interactions promote proliferation while HMW-HA:CD44 interactions suppress proliferation, which can be mediated by an association of CD44 with the tumor suppressor, Merlin (NF2). HMW-HA:CD44 signals result in cell cycle arrest and are one mechanism for reducing susceptibility to cancer. **(B)** Dorsal skin of UVB-exposed keratinocyte tumor-susceptible mice treated topically with HMW-hyaluronan-phosphatidylethanolamine (HA-enriched) do not form tumors, accumulate higher levels of HMW-HA (top panels) and CD44 (bottom panels), and lower levels of hyaluronidases than UVB-irradiated controls. Epidermal HA (brown) is detected via an HA-binding biotinylated protein on paraffin-embedded tissue counterstained with hematoxylin (blue). CD44 (red) and RHAMM (green) are detected using pan-antibodies in dorsal skin of mice counterstained with DAPI (blue).

The naked mole-rat utilizes many tumor resistance mechanisms, including the HMW-HA-regulated mechanism termed “early contact inhibition” (ECI), a robust form of contact inhibition that involves expression of a novel isoform of p16^INK4A^ (105). HA induces early expression at the p16^INK4A^ locus through CD44 binding, resulting in blocked phosphorylation of Rb and attenuation of cell cycle. Naked mole-rat fibroblasts cultured in the presence of hyaluronidase display no ECI and downregulate p16^INK4A^ expression (16). This effect of HMW-HA is mediated by CD44:NF2 signaling (24, 105) (Figure 2A).The growth-inhibiting non-phosphorylated form of NF2 is predominant in cultured naked mole-rat cells, but the addition of hyaluronidase stimulates NF2 phosphorylation, which promotes cell growth (24).

## HMW-HA as a Chemoprevention Strategy

To date, HMW-HA has not been used as a chemoprevention strategy, although oral consumption has been shown to restrict tissue inflammation, notably in the bowel (111). The barrier to topical application of HMW-HA has historically been its restricted passage through the outer cornified layer of the epidermis. However, the recent development of high molecular-weight hyaluronan-phosphatidylethanolamine polymers [E. Turley (2010), Patent number: *US20130059769A1*] that cross the epidermis and form coats around keratinocytes and dermal cells (112) will permit an assessment of whether or not increasing skin HMW-HA can reduce the predisposition of skin to tumorigenesis in cancer-susceptible species (Figure 2B).

## Conclusions

In summary, LMW-HA augments the proliferative and migratory capacities of tumor cells, while HMW-HA reduces tumorigenicity and confers cancer resistance by restricting proliferation, limiting inflammation, neo-angiogenesis, and possibly DNA damage. Further research is required to harvest the full therapeutic potential of targeting LMW-HA polymers and utilizing the tumor resistance properties of HMW-HA. Improved understanding of the mechanisms augmenting the size-dependent biological effects of HA is likely to advance new therapeutic development to limit tumorigenesis.

## Author Contributions

ML provided the first draft of the manuscript, which was edited by ET and CT. CT and ML provided the data for figures. ET assembled all figures presented in this review.

### Conflict of Interest Statement

The authors declare that the research was conducted in the absence of any commercial or financial relationships that could be construed as a potential conflict of interest.
